# Prevalence of cardiovascular diseases and risk factors in adult patients with haemophilia: a cross-sectional study in a tertiary care hospital clinic in Sri Lanka

**DOI:** 10.1186/s12872-022-02789-1

**Published:** 2022-07-30

**Authors:** Thamudika Vithanage, Visaka Ratnamalala, Chandana Wickramaratne, Gaya Katulanda, Chithramali Hasanthika Rodrigo

**Affiliations:** 1 Haematology Unit, National Hospital of Sri Lanka, Colombo 10, Sri Lanka; 2 Department of Pathology, Faculty of Medicine, University of Ruhuna, Galle, Sri Lanka; 3 Chemical Pathology Unit, National Hospital of Sri Lanka, Colombo, Sri Lanka; 4 Postgraduate Institute of Medicine, Colombo, Sri Lanka

**Keywords:** Cardiovascular disease, Haemophilia, Prevalence, Risk factors, Hypertension

## Abstract

**Background:**

Management of cardiovascular disease (CVD) in patients with haemophilia is extremely challenging. Therefore, knowing the prevalence of CVD and risk factors in this population is imperative.

**Methods:**

All consented patients ≥ 18 years of age attending the haemophilia clinic at a tertiary care centre were recruited to the study. Data were collected using a pretested, investigator administered questionnaire. Seated blood pressure, anthropometric measurements and blood samples were obtained using standard techniques. Lipid profile and fasting plasma glucose were estimated. Prevalence of risk factors for CVD was compared with those of age matched males in the general population. P values < 0.05 were considered significant.

**Results:**

Of the total 109 participants, 92 (84.4%) had haemophilia A. The median age of the study group was 36 years. Three (2.8%) had at least one cardiovascular disease. There were 10 (9.2%), 30 (27.5%), 13 (11.9%) and 4 (3.7%) participants with diabetes, hypertension, current smoking and obesity (Body Mass Index (BMI) ≥ 30 kg/m^2^) respectively. 32 (29.4%) and 37 (33.9%) participants had waist circumference ≥ 90 cm and waist hip ratio ≥ 0.9 respectively. 38 (34.9%) had total cholesterol ≥ 200 mg/dl, 43 (39.5%) had low density lipoprotein (LDL) cholesterol ≥ 130 mg/dl, 25 (22.9%) had triglycerides (TG) ≥ 150 mg/dl and 58 (53.2%) had High density lipoprotein (HDL) cholesterol < 40 mg/dl. Diabetes was significantly associated with factor levels below 5% (p = 0.038). BMI, waist circumference and dyslipidaemia in the study were significantly higher compared to the general population.

**Conclusion:**

The study signifies an increased prevalence of risk factors for CVD among patients with haemophilia and the need for preventive measures.

## Background

Haemophilia is a rare X linked recessive inherited bleeding disorder. [1]. Factor levels < 1%, 1–5% and > 5% are considered severe, moderate and mild respectively [2]. Over the past few decades, treatment of haemophilia has seen major advances thus the quality of life as well as the life expectancy of patients have increased [3–6]. With more patients living up to an older age, emergence of age-related comorbidities such as CVD in them have raised a concern [6]. As the treatment modalities for CVD are based on blood thinning and antiplatelet effects, management of this group of patients has proved to be quite challenging due to their inherent bleeding tendency [7].

Hypertension, diabetes mellitus, dyslipidaemia, smoking and obesity are well known modifiable risk factors for CVD. Abdominal obesity in particular, is evidenced to exacerbate other risk factors and promote CVD [8]. Existing data suggest that the prevalence of these risk factors in patients with haemophilia is variable when compared with the general population [9–13].

Although traditionally it was believed that patients with haemophilia have a low risk for arterial thromboses due to their intrinsic hypocoagulable state, recent research data looking at the prevalence of CVD have shown mixed results [9, 11–13]. Several studies have demonstrated an increased risk of CVD and its risk factors in patients with haemophilia compared to the general population [9, 10].

In Sri Lanka, comprehensive care for patients with haemophilia has improved greatly over the years in keeping with the global trends. This study was performed with the aim of obtaining, prevalence data for CVD and risk factors among adult haemophilia patients since it is a timely need. Also, we aimed to compare the prevalence data with the existing data of the general population of the same gender, to find out whether there are any significant variations that might be resulting from the background coagulopathy.

## Materials and methods

This descriptive cross-sectional study was carried out in the largest haemophilia center in Sri Lanka after obtaining ethical approval for the study from the Ethics Review Committee, Faculty of Medicine, University of Colombo, Sri Lanka (FERCAP accredited). All the patients ≥ 18 years of age attending the hemophilia clinic and consented were recruited to the study. Patients with uncertain factor levels and carrier women were excluded from the study. Socio-demographic data (age, race, religion, current employment and monthly family income), data on CVD events (coronary artery disease, non haemorrhagic stroke and transient ischaemic attack) and risk factors (diabetes, hypertension, dyslipidaemia and smoking status) were collected using a pre-tested, interviewer administered questionnaire and clinical records.

Height, waist and hip circumferences were measured to the nearest centimeter according to standard methods. Weight was measured into the nearest gram using standard methods [14, 15]. The BMI was calculated as the body weight in kilograms divided by the height in meters squared. The waist hip ratio was obtained by dividing waist circumference by hip circumference. After relaxing for 5–10 min, seated blood pressure (BP) was measured using a validated mercury sphygmomanometer using standard technique [16]. BP was measured twice with a gap of five minutes and averaged.

Venous blood samples were collected for fasting plasma glucose and lipid profile using standard techniques after an overnight fast of 12 h from the patients who were not diagnosed of having diabetes or dyslipidaemia respectively [17]. Samples were analyzed using a fully automated analyzer (Abbott architect c8000 biochemistry). The assay methods have been validated and appropriate quality control measures were applied to ensure reliability of results.

Participants were considered to have hypertension if they had been previously diagnosed (verified through previous medical records and anti-hypertensive prescriptions) or if the average of two resting seated BP readings, separated by 5 min were ≥ 140/90 mmHg [18]. Patients were considered to have diabetes if they had been previously diagnosed (verified through previous medical records, laboratory reports and prescriptions) or by having a fasting venous plasma glucose ≥ 126 mg/dl (World Health Organization and American Diabetes Association criteria) [19, 20]. Lipid profiles were categorized as normal and high according to National Cholesterol Education Programme/Adult Treatment Panel III (NCEP/ATP III) criteria [21].

The data were analyzed with SPSS 20 software. Prevalence of CVD and risk factors were determined by calculating the proportions and were compared with that of the general population. The significance of the differences between proportions (%) and means were tested using χ2-test and Student’s t-test, z test for proportions respectively. P values < 0.05 were considered significant.

## Results

The total number of participants was 109 and the mean age of the study group was 37.7 ± SD 12.8 years. Majority (76.1%, n = 83) of the participants were employed and of 73.5% the monthly family income was > 10 000 LKR (50 USD) (Table 1).

**Table 1:** Frequency distribution of the selected socio-demographic characteristics of the participants (N = 109)

Characteristic	Number	%
**Age**		
≤ 50 years	94	86.2
> 50 years	15	13.8
**Race**		
Sinhala	92	84.4
Muslim	8	8.3
Tamil	9	7.3
**Religion**		
Buddhism	84	77.1
Hindu	7	6.4
Islam	9	8.3
Christian/Catholic	9	8.3
**Current employment**		
Employed	83	76.1
Unemployed	9	8.3
Student	15	13.8
Retired	2	1.8
**Family income**		
≤ 10,000 (Rs)	29	26.6
10,001–50,000 (Rs)	66	60.6
50,001–100 000 (Rs)	11	10.1
> 100,000 (Rs)	3	2.8

Majority of the participants (84.4%, n = 92) had hemophilia A, and 51.4% (n = 56) had factor levels < 1%. Most of the participants (57.8%, n = 63) were on prophylaxis, while inhibitors had never been detected in 57.8% (n = 63) participants (Table 2).

**Table 2:** Characteristics related to hemophilia (N = 109)

Characteristic	Number	%
**Type**		
A	92	84.4
B	17	15.6
**Factor level**		
< 1%	56	51.4
1–5%	37	33.9
5–40%	16	14.7
**Prophylaxis**		
Yes	63	57.8
No	46	42.2
**Inhibitor status**		
Positive	15	13.8
Never detected	89	81.7
Transient	4	3.7
On ITI	1	0.9

The prevalence of at least one diagnosed CVD among the participants was 2.8% (n = 3) (Table 3).

**Table 3:** Prevalence of cardiovascular diseases among the participants (N = 109)

	Angina	Myocardial infarction	Undergone CABG or coronary stenting	Transient ischemic attacks (TIA)	Non hemorrhagic stroke	At least one CVD
Number of patients	3	3	3	1	1	3
%	2.8	2.8	2.8	0.9	0.9	2.8

Majority (53.2%) had HDL cholesterol < 40 mg/dl as a risk factor for CVD. The prevalence of risk factors in the study group is given in Table 4.

**Table 4:** Prevalence of selected risk factors for cardiovascular diseases among the participants (N = 109)

Risk factor	Number of patients	%
Diabetes	10	9.2
Hypertension	30	27.5
Current smoking	13	11.9
Obesity (BMI > 30 kg/m^2^) ^†^	4	3.7
Overweight (BMI 25–29.9 kg/m^2^) ^†^	39	35.8
Waist circumference ≥ 90 cm †	32	29.4
Waist hip ratio ≥ 0.9 †	37	33.9
Total cholesterol ≥ 200 mg/dl ‡	38	34.9
LDL cholesterol ≥ 130 mg/dl ‡	43	39.5
Triglycerides ≥ 150 mg/dl ‡	25	22.9
HDL cholesterol < 40 mg/dl ‡	58	53.2

^†^World Health Organization defined cut off values[22]

^‡^Categories according to National Cholesterol Education Programme/Adult Treatment Panel III guidelines[21]

Prevalence of selected risk factors for CVD in the study group show significantly higher BMI, waist circumference and dyslipidaemia (*p* < 0.00001) compared to the age matched males (Table 5).

**Table 5:** Comparison of the prevalence of selected risk factors for cardiovascular diseases in the study with the general population

Risk factor	Study	Sri Lanka diabetes and cardiovascular study [23]	P*
Diabetes (%)	9.2	11.3	*p* = 0.490
Hypertension (%)	27.5	27.1	*p* = 0.912
Current smoking (%)	11.9	38.6	P < 0.00001
BMI (kg/m^2^)	23.4 ± 3.8	21.1 ± 3.7	*p* < 0.00001
Waist circumference (cm)	83.9 ± 11.1	78.1 ± 11	*p* < 0.00001
Dyslipidemia (%)	90.8	73.5	*p* < 0.00001

*p < 0.00001

Of all the CVD risk factors, only diabetes was significantly associated with the severity of haemophilia (Table 6).

**Table 6:** Association between severity of haemophilia and risk factors for cardiovascular diseases

Risk factor	Factor level				P*
	< 5%		5–40%		
	N	%	N	%	
Diabetes	6	60.0	4	40.0	*p* = 0.038
Hypertension	25	83.3	5	16.7	*p* = 0.765
Obesity (BMI > 30 kg/m^2^)	57	86.4	9	13.6	*p* = 0.126
Waist circumference ≥ 90 cm	28	87.5	4	12.5	*p* = 0.774
Dyslipidemia	84	84.8	15	15.2	*P* = 1.000

*p < 0.05

## Discussion

In this study, the prevalence of CVD and its risk factors in patients with haemophilia aged 18 and above in the largest treatment centre of Sri Lanka was assessed and compared with available data for the general population. Both haemophilia A and B patients were included in the study irrespective of their factor levels, inhibitor status or whether on prophylaxis. Majority of the participants (N = 94, 86.2%) in the study group were below 50 years of age. This reflects inclusion of younger patients in the prophylaxis program. In keeping with global statistics, majority (84%) of participants were having haemophilia A. Higher proportion (85.3%, n = 93) of the participants had either severe or moderate haemophilia.

Even though it is hypothesized that the severe form of haemophilia is protective against thrombotic events, existing literature has conflicting evidence on this [9–13]. Knowing prevalence of CVD and its risk factors among patients with haemophilia would enable implementing timely preventive measures.

In our study, there were three patients (2.8%) with coronary heart disease and one patient with non haemorrhagic stroke. The participants with TIA and stroke also had coronary events in the past. Studies conducted to assess the prevalence of cardiovascular diseases in adult general population in Sri Lanka show prevalence of coronary heart disease in 16/1000 and prevalence of stroke and risk factors for stroke in /1000 although these are not directly comparable to the current study [24, 25].

Comparison of CVD risk factors of the study population with the data stated for males (n = 1758) in Sri Lanka diabetes and cardiovascular study (SLDCS) carried out in healthy participants who were aged ≥ 18 years shows that, dyslipidaemia, BMI and waist circumference were significantly higher among patients with haemophilia, than normal healthy adults [23]. According to SLDCS, prevalence of dyslipidaemia in Sri Lanka exceeds most of the regional and non-Asian countries. Further, it states that “Sri Lankans have a unique pattern of dyslipidaemia with lower HDL cholesterol, higher triglycerides and higher LDL cholesterol”. In our study, the prevalence of high LDL, TG and low HDL were 39.5%, 22.9% and 53.2% respectively, in keeping with the pattern described in the SLDCS.

In the retrospective study carried out by Ming Y. Lim et al. in 58 haemophilia patients attending the Mayo Comprehensive Haemophilia Clinic during 2006–2009 period, the prevalence of CVD risk factors was analyzed and compared with the existing data for the general population. They have found a higher prevalence of hypertension and a lower prevalence of current smoking and obesity. Diabetes in the sample was similar in prevalence to the general population statistics while they could not assess the prevalence of dyslipidaemia due to missing data [26].

In a large US study (J. Pocoski et al. 2013), when compared to age-matched males in the general population, 2506 patients with haemophilia were found to have a significantly higher prevalence of coronary artery disease (10.7% vs 5.8%, P < 0.001), hypertension (22.6% vs 15.5%, P < 0.001) and hyperlipidaemia (15.9% vs 11.9%, P = 0.001). Also, they found that cardiovascular comorbidities in patients with haemophilia occurred at an earlier age than the general population. However, their study did not assess the prevalence of other CVD risk factors like obesity, diabetes, family history and smoking status and they have included only patients with haemophilia A. They have hypothesized that similar to other chronic inflammatory disorders, incidence of CVD might have increased in patients with haemophilia as they have chronically inflamed joints as a result of recurrent joint bleeds [10].

In a second retrospective database analysis, Pocoski J et al. were able to confirm the previous study findings of increased cardiovascular comorbidities in patients with haemophilia A in the US [27].

Significantly increased prevalence of obesity, increased waist circumference and dyslipidemia in the current study might have resulted from limited mobility and lack of proper physical exercise due to the fear of bleeding. Interestingly, the smoking habits were significantly lower in the study population compared to normal healthy adult males in Sri Lanka [23].

The significant association of diabetes mellitus observed in participants with severe haemophilia in the current study urge further studies to ascertain underlying causative factors. An increased prevalence of hypertension in patients with haemophilia was not evident in this study even though it was shown in several studies [10, 26, 28, 29].

The main limitation in this study is the lack of a control group. In addition, although the prevalence of CVD in patients with haemophilia was calculated, it could not be compared with the healthy adult population of the country due to lack of a matched control group. Very low number of patients with actual CVD has made analysis of associations with factor levels and inhibitor status invalid. Also, there is a possibility of missing patients with actual CVD but no documentation, as we have considered only those with a clearly documented history of CVD as positive.

## Conclusions and recommendations

The prevalence of diabetes mellitus, dyslipidaemia, increased BMI, waist circumference and cardiovascular events were substantially high among patients with haemophilia. This stresses the need for screening and active measures to control these risk factors to prevent cardiovascular events in patients with haemophilia. Further research using larger sample size and active control groups are recommended to validate these findings.

## Data Availability

The datasets used and/or analyzed during the current study are available from the corresponding author on reasonable request.

## Abbreviations

BMI: Body mass index
BP: Blood pressure
CABG: Coronary Artery Bypass Grafting
CVD: Cardiovascular disease
HDL: High Density Lipoprotein
FERCAP: Forum for Ethical Review Committees in the Asian and Western Pacific Region
LDL: Low Density Lipoprotein
LKR: Sri Lankan Rupees
NCEP/ATP III: National Cholesterol Education Programme/Adult Treatment Panel III
NHSL: National Hospital of Sri Lanka
SLDCS: Sri Lanka diabetes and cardiovascular study
TG: Triglycerides
TIA: Transient Ischaemic Attack
USD: United States Dollar

## References

[1] Srivastava A, Santagostino E, Dougall A (2020). WFH guidelines for the management of hemophilia. Haemophilia.

[2] White GC, Rosendaal F, Aledort LM (2001). Definitions in hemophilia: recommendation of the Scientific Subcommittee on Factor VIII and Factor IX of the Scientific and Standardization Committee of the International Society on Thrombosis and Haemostasis. Thromb Haemost.

[3] Ling G, Nathwani AC, Tuddenham EG (2018). Recent advances in developing specific therapies for haemophilia. Br J Haematol.

[4] Pasi KJ, Rangarajan S, Mitchell N (2020). Multiyear follow-up of AAV5-hFVIII-SQ gene therapy for hemophilia A. N Engl J Med.

[5] Franchini M, Mannucci PM (2014). The history of hemophilia. Semin Thromb Hemost.

[6] Shapiro S, Makris M (2019). Haemophilia and ageing. Br J Haematol.

[7] Cayla G, Morange PE, Chambost H (2013). Management of cardiovascular disease in haemophilia. Thromb Res.

[8] Yusuf S, Hawken S, Ôunpuu S (2004). Effect of potentially modifiable risk factors associated with myocardial infarction in 52 countries (the INTERHEART study): case–control study. Lancet.

[9] Sharathkumar AA, Soucie JM, Trawinski B (2011). Prevalence and risk factors of cardiovascular disease (CVD) events among patients with haemophilia: experience of a single haemophilia treatment centre in the United States (US). Haemophilia.

[10] Pocoski J, Ma A, Kessler CM (2014). Cardiovascular comorbidities are increased in U.S. patients with haemophilia A: a retrospective database analysis. Haemophilia.

[11] Sood SL, Cheng D, Ragni M (2018). A cross-sectional analysis of cardiovascular disease in the hemophilia population. Blood Adv.

[12] Biere-Rafi S, Baarslag MA, Peters M (2011). Cardiovascular risk assessment in haemophilia patients. Thromb Haemost.

[13] Rizwan I, Minuk L, Jackson S (2015). Cardiovascular disease prevalence and relevance in haemophilia: a scoping review. Haemophilia.

[14] Jelliffe DB (1966). The assessment of the nutritional status of the community (with special reference to field surveys in developing regions of the world). Monogr Ser World Health Organ.

[15] World Health Organization. Waist circumference and waist-hip ratio: report of a WHO expert consultation, Geneva, 8–11 December 2008.

[16] Smith L (2005). New AHA recommendations for blood pressure measurement. Am Fam Phys.

[17] World Health Organization (2010). WHO guidelines on drawing blood: best practices in phlebotomy.

[18] Chobanian AV, Bakris GL, Black HR (2003). Seventh report of the joint national committee on prevention, detection, evaluation, and treatment of high blood pressure. Hypertension.

[19] WHO. Definition, diagnosis and classification of diabetes mellitus and its complications. Report of a WHO Consultation. Part 1. Diagnosis and Classification of Diabetes Mellitus. Document number WHO/NCD/NCS/99.2. Geneva: World Health Organization; 1999.

[20] American Diabetes Association (1997). Report of the expert committee on the diagnosis and classification of diabetes mellitus. Diabetes Care.

[21] Grundy SM, Cleeman JI, Daniels SR (2005). Diagnosis and management of the metabolic syndrome: an American Heart Association/National Heart, Lung, and Blood Institute Scientific Statement. Circulation.

[22] World Health Organization Expert Consultation (2004). Appropriate Body Mass Index (BMI) for Asian populations and its implication for policy and intervention strategies. Lancet.

[23] Katulanda P, Dissanayake HA, De Silva SN (2018). Prevalence, patterns, and associations of dyslipidemia among Sri Lankan adults—Sri Lanka Diabetes and Cardiovascular Study in 2005–2006. J Clin Lipidol.

[24] Mendis S, Ekanayake EM (1994). Prevalence of coronary heart disease and cardiovascular risk factors in middle aged males in a defined population in central Sri Lanka. Int J Cardiol.

[25] Chang T, Gajasinghe S, Arambepola C (2015). Prevalence of stroke and its risk factors in urban Sri Lanka: population-based study. Stroke.

[26] Lim MY, Pruthi RK (2011). Cardiovascular disease risk factors: prevalence and management in adult hemophilia patients. Blood Coag Fibrinol.

[27] Humphries TJ, Ma A, Kessler CM (2016). A second retrospective database analysis confirms prior findings of apparent increased cardiovascular comorbidities in hemophilia A in the United States. Am J Hematol.

[28] Barnes RF, Cramer TJ, Sait AS (2016). The hypertension of hemophilia is not explained by the usual cardiovascular risk factors: results of a cohort study. Int J Hypertens.

[29] Barnes RF, Cramer TJ, Hughes TH (2017). The hypertension of hemophilia is associated with vascular remodeling in the joint. Microcirculation.

